# Seed-mediated growth of MOF-encapsulated Pd@Ag core–shell nanoparticles: toward advanced room temperature nanocatalysts†
†Electronic supplementary information (ESI) available: Experimental details and catalysts characterization. See DOI: 10.1039/c5sc02925b



**DOI:** 10.1039/c5sc02925b

**Published:** 2015-09-23

**Authors:** Liyu Chen, Binbin Huang, Xuan Qiu, Xi Wang, Rafael Luque, Yingwei Li

**Affiliations:** a School of Chemistry and Chemical Engineering , South China University of Technology , Guangzhou 510640 , China . Email: liyw@scut.edu.cn; b Departamento de Química Orgánica , Universidad de Córdoba , Edif. Marie Curie, Ctra Nnal IV-A, Km 396 , E14014 , Córdoba , Spain . Email: q62alsor@uco.es

## Abstract

The possibility of using inner cavities within metal–organic frameworks (MOFs) as templates for the fabrication of tiny metal nanoparticles (NPs) was attempted in this work. An unprecedented design of Pd@Ag core–shell NPs on MOFs *via* a seed mediated growth strategy is reported and attributed to the presence of activated physisorbed hydrogen atoms on embedded Pd NPs as reducing agents to selectively direct the deposition of Ag onto Pd while minimizing the Ag self-nucleation. The obtained Pd@Ag core–shell NPs exhibited a significant increase in selectivity in the partial hydrogenation of phenylacetylene as compared to their monometallic counterparts, due to the surface dilution and electron modification of the surface Pd sites by Ag deposition. Pd@Ag NPs also possessed an unprecedented high stability and recyclability in the catalytic reactions, related to the nano-confinement effect and the strong metal–support interaction offered by the MOF framework.

## Abstract 2

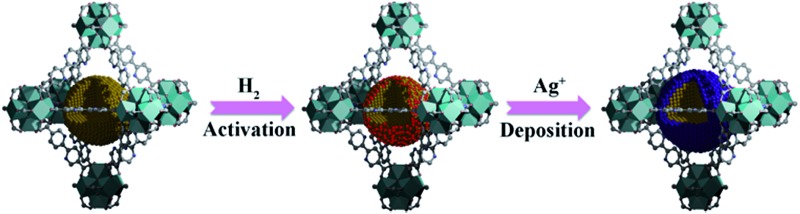
Core–shell Pd@Ag nanoparticles are formed within the pores of MOFs *via* a seed mediated growth strategy with activated hydrogen atoms as the reducing agent, leading to a family of bimetallic core–shell MOF nanomaterials with excelling catalytic performance in room temperature reactions.

## Introduction

Bimetallic core–shell nanoparticles (NPs) have attracted increasing attention in recent years because of their fascinating properties and applications in a variety of fields, especially in catalysis.^1^ The inner core metal can significantly influence the external shell of another metal to provide unique physico-chemical properties.^2^ These properties can be tuned by controlling the size, composition and structure of bimetallic core–shell NPs. In this regard, template-directed syntheses were demonstrated as effective strategies to control the growth of metal NPs by the pore size, shape and channel structure of porous materials (*e.g.*, zeolites, mesoporous silicas and carbon nanotubes).^3^ Nevertheless, few attempts were reported to date on the preparation of ultrasmall bimetallic core–shell NPs within several nanometers using porous materials as templates.^4^

Metal–organic frameworks (MOFs) emerged as a new class of porous crystalline materials featuring tunable pore structures, large internal surface areas and promising functionalities.^5^ These properties make MOFs very appropriate candidates for the stabilization of metal NPs.^6^ The nodal metal ions and aromatic backbone of MOFs can particularly interact weakly with embedded metal NPs through coordination and π–π forces, which can lead to charge transfers to such NPs to trigger an enhanced stability/activity performance.^7^ The possibility of using the inner cavities of MOFs as templates for the design of tiny metal/metal oxide NPs can be therefore considered of high relevance in the design of advanced nanocatalysts.^8^ Previous studies proved the successful incorporation of monometallic or bimetallic alloy NPs within the pores of MOFs.^9^ However, the incorporation of bimetallic core–shell NPs into the MOF pores remains a great challenge as the core–shell NPs would be more easily deposited on the external surface of MOFs.^4a,b^ Such difficulties come from the inherent MOF microstructures which make it difficult for the structure and composition tailoring of the embedded species in the restricted space to occur. To the best of our knowledge, there is only one example to date claiming the preparation of Pd@Co core–shell NPs within MOFs by a simultaneous reduction method.^4*c*^ Nevertheless, the simultaneous reduction method is not widely employed for core–shell NPs as it produces alloyed NPs in most cases.^1*b*^

Seed-mediated growth has been well-documented as the most powerful route among the developed synthetic methods to synthesize bimetallic core–shell NPs.^10^ In a typical seed mediated growth method, pre-formed seeds of one metal serve as nucleation sites for the further growth of another metal. During the subsequent reductive growth of the metal shell, a sophisticated and careful control is required to avoid the individual nucleation and growth of the secondary metal as individual particles.

**Scheme 1:**
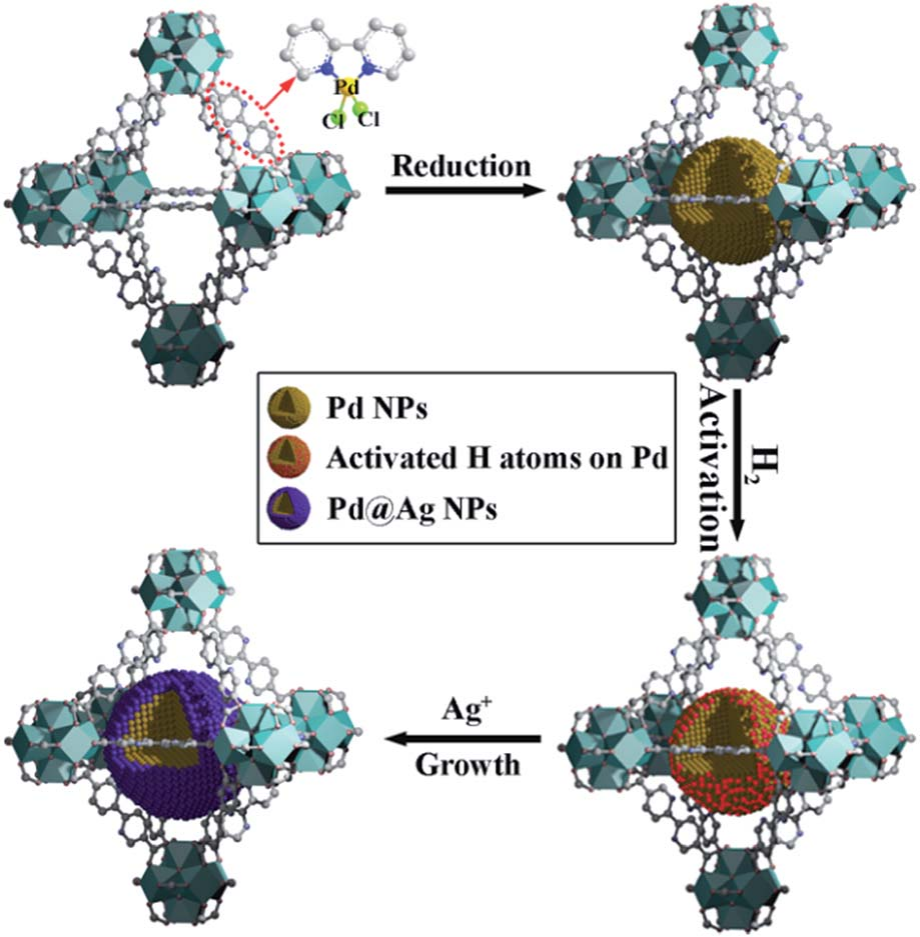
Schematic illustration of the fabrication of Pd@Ag core–shell NPs encapsulated in the MOF pore.

Herein, we report a simple, efficient and unprecedented approach for the preparation of ultrafine Pd@Ag core–shell NPs within the pores of a MOF under a seed-mediated growth strategy with activated hydrogen atoms as the reducing agent. This strategy involves a multistep approach, (1) first is the encapsulation of Pd NPs within the MOF through a pre-incorporation method followed by (2) dissociation and activation of hydrogen molecules on the surface of Pd NPs to serve as effective reducing agents for (3) a selective deposition of Ag on Pd (Scheme 1). The success of the proposed strategy can be ascribed to the activated hydrogen atoms confined on the embedded Pd NP surfaces, which promoted the exclusive reduction of Ag^+^ on Pd and prevented a significant self-nucleation of Ag to generate individual Ag NPs. Such a rational design was demonstrated to provide the encapsulated ultrafine Pd@Ag core–shell NPs with various Pd/Ag ratios and average sizes of *ca.* 2.6–3.1 nm within the MOF pores. Interestingly, the Ag shell could effectively block the high coordination sites on the Pd core, thus leading to a significant increase in the selectivity in the partial hydrogenation of phenylacetylene, selected as the model reaction.

## Results and discussion

UiO-67 MOF, based on Zr_6_O_4_(OH)_4_(CO_2_)_12_ secondary building units (SBUs) and dicarboxylate bridging ligands, was selected as the host matrix for the encapsulation of NPs due to its high physicochemical stability and tunable functionalities of ligands as well as large BET surface area (up to 2000 m^2^ g^–1^).^11^ In our previous work, we have developed a pre-incorporation strategy to exclusively encapsulate Pd NPs within the pores of a MOF, preventing any external surface deposition.^9*a*^ We also demonstrated that hydrogen could be dissociated into atomic hydrogen and spillover onto the surface of Pd– or Pt–MOF composites.^12^ The highly active H atoms possessed a remarkably superior reducing ability as compared to H_2_ to effectively reduce Ag^+^ to metallic Ag, otherwise unachievable using molecular hydrogen at room temperature.^13^

In a typical synthesis of Pd@Ag-in-UiO-67, Pd precursors were first incorporated within UiO-67 using a pre-incorporation strategy (see ESI† for details), followed by treatment under H_2_ at 200 °C for 2 h to yield Pd-in-UiO-67. Pd-in-UiO-67 was subsequently dispersed in DMF and the solution was bubbled with H_2_ for 1 h at room temperature followed by the addition of a AgNO_3_ solution under stirring, to promote the growth of Ag on the surface of the embedded Pd NPs.

As measured by atomic absorption spectroscopy (AAS), the Pd/Ag molar ratio in the as-prepared Pd@Ag-in-UiO-67 was calculated to be 1/1.4. Powder X-ray diffraction (PXRD) patterns of UiO-67 and metal-loaded materials are presented in Fig. S1.† The incorporation of metals in UiO-67 did not cause any apparent loss of crystallinity, indicating that the MOF structure was mostly preserved upon metal incorporation. No identifiable peaks for metal NPs were observed most probably due to the low metal content in the materials. Porosity is one of the key factors that determines the ability of porous materials for catalytic applications. N_2_ adsorption/desorption experiments (Fig. S2†) of UiO-67 before and after loading of metal NPs exhibited a mixture of type I and IV curves. The surface areas obtained were 2415, 2101, and 1893 m^2^ g^–1^ for the as-synthesized UiO-67, Pd-in-UiO-67, and Pd@Ag-in-UiO-67 (1 : 1.4), respectively (Table S1†). The observed lower surface areas of the UiO-67-encapsulated metal samples with respect to those of the parent UiO-67 indicated that the cavities of UiO-67 could be partially occupied by metal NPs.

**Fig. 1:**
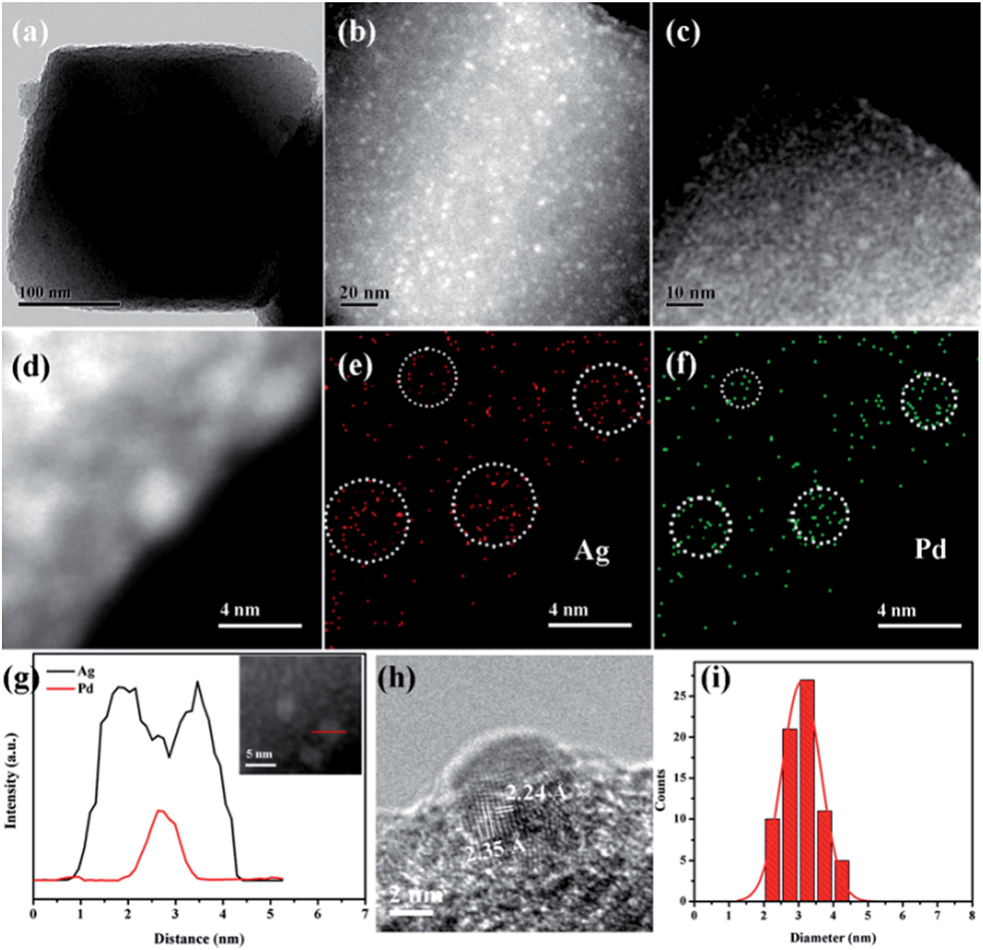
TEM image of Pd@Ag-in-UiO-67 (1 : 1.4) (a). HAADF-STEM image of ultrathin cuts from Pd@Ag-in-UiO-67 (1 : 1.4) (b). HAADF-STEM image of Pd-in-UiO-67 (c). HAADF-STEM image of ultrathin cuts from Pd@Ag-in-UiO-67 (1 : 1.4) (d) and the corresponding STEM-EDX elemental mapping images of Ag and Pd (c–f). Elemental line-scanning spectra of Pd@Ag-in-UiO-67 (1 : 1.4) (inset) along the direction marked by a red line, which unambiguously confirmed the formation of core–shell NPs (g). HRTEM image of a Pd@Ag NP (h). The corresponding size distribution of Pd@Ag core–shell NPs (i).

The morphology and structure of Pd-in-UiO-67 and Pd@Ag-UiO-67 (1 : 1.4) were investigated by transmission electron microscopy (TEM), high-angle annular dark-field scanning transmission electron microscopy (HAADF-STEM), and energy-dispersive X-ray spectroscopy (EDS) elemental mapping analyses. Pd-in-UiO-67 contained a homogeneous distribution of Pd NPs within UiO-67, with an average size of 2.5 nm (Fig. 1c). HAADF-STEM images of ultrathin slices from Pd-in-UiO-67 suggested that Pd NPs were mostly located inside UiO-67 (Fig. S3†). After Ag growth onto Pd NPs, the obtained Pd@Ag NPs possessed an average particle size of 3.1 ± 0.5 nm, featuring an excellent dispersion in UiO-67 with an octahedral shape (Fig. 1a). Note that the particle sizes somewhat exceeded the pore size of the MOF. Such a phenomenon might be interpreted by the local defects/deformations of the host frameworks by the growth of metal NPs (MNPs), which is in accordance with other MNPs@MOF composites.^4a,9a^ The HAADF-STEM image of the ultrathin micrometer-sized slices of Pd@Ag-in-UiO-67 further confirmed the presence of Pd@Ag NPs homogeneously distributed within the MOF framework (Fig. 1c). Although the Pd@Ag core–shell structure was difficult to distinguish from HAADF-STEM images due to the very close atom masses between Pd and Ag, the structure could be unambiguously demonstrated by the EDS mapping and line profile analysis. As shown in Fig. 1d–g, the element Pd was distributed only in the core with an Ag-rich shell, suggesting that the growth of Ag exclusively took place on the Pd surface. More importantly, no isolated Ag NPs could be visualized in the materials. A representative high-resolution TEM (HRTEM) image of a deliberately selected large Pd@Ag NP showed that the interplanar spacings of the particle lattices were 0.224 and 0.235 nm, corresponding to the (111) lattice spacing of the face-centered cubic Pd and cubic Ag, respectively (Fig. 1h).

**Fig. 2:**
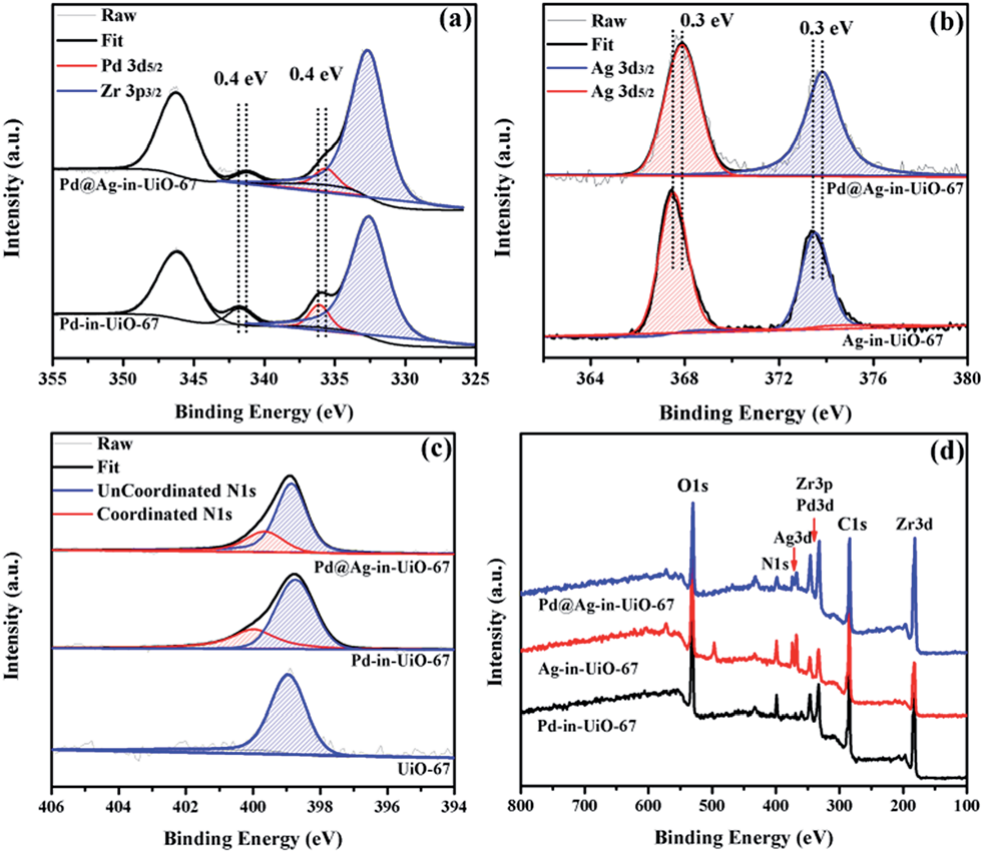
XPS spectra of Pd-in-UiO-67, Ag-in-UiO-67, and Pd@Ag-in-UiO-67 (1 : 1.4) referenced to the hydrocarbon C 1s. (a) Pd 3d, (b) Ag 3d, (c) N 1s, and (d) survey photo emission spectrum.

X-Ray photoelectron spectroscopy (XPS) experiments with Ar etching were subsequently conducted to further confirm the structural and electronic environment of the core–shell NPs. XPS data showed that the Pd 3d 5/2 peak for Pd@Ag-in-UiO-67 (1 : 1.4) was located at around 335.7 eV, corresponding to the zero-valent Pd species. As compared to the monometallic Pd-in-UiO-67, the binding energy was interestingly shifted to a lower value by approximately 0.4 eV (Fig. 2a). In the case of Pd@Ag-in-UiO-67, the observed Ag 3d 5/2 binding energy at 367.9 eV clearly corresponds to Ag^0^, observed at higher binding energies with respect to Ag-in-UiO-67 (Fig. 2b). These results pointed to a modification of the electronic structure of Pd and Ag atoms, further confirming the formation of bimetallic NPs. XPS of Pd@Ag-in-UiO-67 before and after the Ar etching also revealed an Ag-rich shell and a Pd core (Fig. S4†). Additionally, a new N 1s peak at *ca.* 400 eV was observed for UiO-67-encapsulated metal samples as compared to the N 1s peak in the pristine UiO-67 (Fig. 2c). Such a N 1s shift towards higher binding energies was attributed to the slight transfer of electrons to the embedded metal NPs. Such an electron transfer observed from the XPS spectra would support the fact that Pd@Ag NPs possessed certain chemical interactions with the MOF support.

The synthetic procedure could be readily extended to the preparation of Pd@Ag-in-UiO-67 materials with different Pd/Ag ratios. As Pd-in-UiO-67 was allowed to bubble with excess H_2_, surface Pd atoms were expected to be (almost) fully covered by activated H atoms. Each H atom donated one electron for the reductive deposition of Ag^+^ on Pd. By varying the amount of AgNO_3_ added into the synthesis solution, the Pd/Ag ratio in the obtained Pd@Ag-in-UiO-67 could be simply controlled until Pd was fully covered. Pd to Ag ratios in the obtained Pd@Ag NPs could be decreased from 30/1 to 1/1.4 when the Ag amount added to the synthesis system was increased from 30 μg to 2 mg as determined by the AAS analysis (Table S2†). Interestingly, a further increase in the Ag amount could maintain 1/1.4 Pd/Ag ratios, implying that Pd was fully covered by Ag. TEM images clearly proved that the average size of Pd@Ag NPs increased from 2.6 to 3.1 nm as the Pd/Ag ratio decreased from 30/1 to 1/1.4 (Fig. S5†). This indicated that Pd NPs were decorated with separated Ag atoms (at low Ag loadings) as the size of the obtained Pd@Ag was similar to that of pure Pd NPs. Ag atoms would then entirely cover the Pd surface at higher Ag loadings (*e.g.*, Pd/Ag = 1 : 1.4).

After detailed characterization, as-prepared Pd and Pd@Ag NPs confined in UiO-67 were explored as catalysts for the partial-hydrogenation of phenylacetylene. The catalytic partial-hydrogenation of alkynes to alkenes is one of the most important transformations in petrochemistry and industry.^14^ However, the challenge to achieve high selectivities to alkenes at high conversions (due to the easy over-hydrogenation of alkenes to undesired alkanes) still remains to date.

**Fig. 3:**
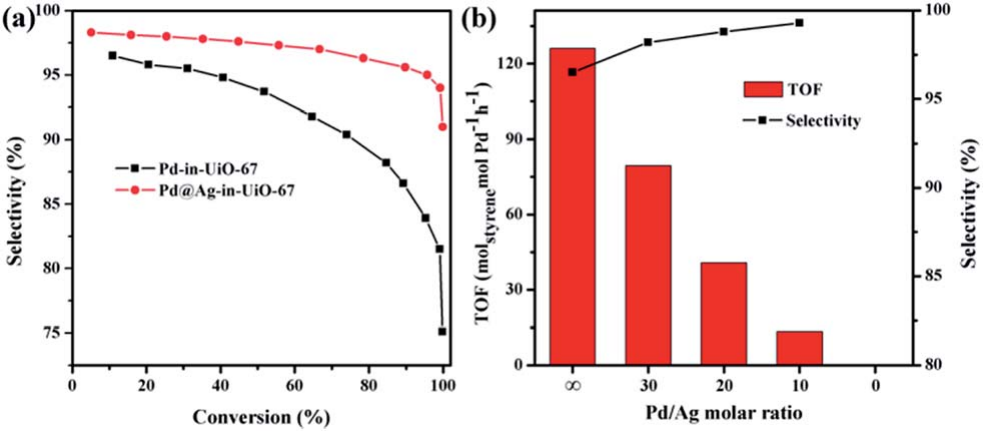
(a) Selectivity to styrene as a function of the phenylacetylene conversion for the reaction over Pd-in-UiO-67 and Pd@Ag-in-UiO-67 (30 : 1). (b) Plot of the TOF value and selectivity to styrene (calculated at 10% conversion of phenylacetylene) *versus* the Pd/Ag molar ratio in Pd@Ag-in-UiO-67. TOF: moles of phenylacetylene produced on per mole of Pd per hour.

All reactions were performed at room temperature and atmospheric hydrogen pressure. The influence of the phenylacetylene conversion on the styrene selectivity was monitored using Pd-in-UiO-67 and Pd@Ag-in-UiO-67 (30 : 1) catalysts. For both catalysts, styrene selectivity generally declined with an increase in the phenylacetylene conversion. However, Pd@Ag-in-UiO-67 exhibited an apparently improved selectivity (91%, quantitative phenylacetylene conversion) as compared to monometallic Pd NPs confined in UiO-67 (75% selectivity, >99% phenylacetylene conversion, Fig. 3a). Importantly, an increase in the Ag loading led to a significant enhancement in the styrene selectivity, accompanied by a gradual loss in the activity (Fig. 3b and S6†). With no catalytic activity observed for Ag-in-UiO-67 under the investigated reaction conditions, the observed decrease in the activity after the Ag deposition can be clearly correlated to the blocking of active surface Pd sites by a Ag shell, confirming the formation of Pd@Ag systems. Pd@Ag-in-UiO-67 (1 : 1.4) provided no catalytic activity under the investigated conditions, further confirming that the Pd surface was fully covered by Ag.

The enhanced selectivity of Pd@Ag-in-UiO-67 as compared to Pd-in-UiO-67 may be attributed to the surface dilution and electron modification effects of Ag on the surface Pd sites. For monometallic Pd NPs, the presence of adjacent Pd sites in large Pd ensembles could simply favor a full hydrogenation of styrene to ethylbenzene. Interestingly, the presence of Ag in the Pd@Ag NPs can originate the Pd surface dilution and result in isolated Pd sites, which could suppress over hydrogenation of phenylacetylene, which is of crucial importance for the selective production of styrene.^15^ The noticeable improvements in the selectivity can also be attributed to a modification of the electronic structure of surface Pd owing to its interaction with Ag, in which a charge transfer Ag-to-Pd was present (as demonstrated by the XPS analysis). The formation of electronegative Pd species could lead to a decrease in the ratio of phenylacetylene/styrene adsorption energies, thus suppressing over-hydrogenation of phenylacetylene.^15*a*^

**Fig. 4:**
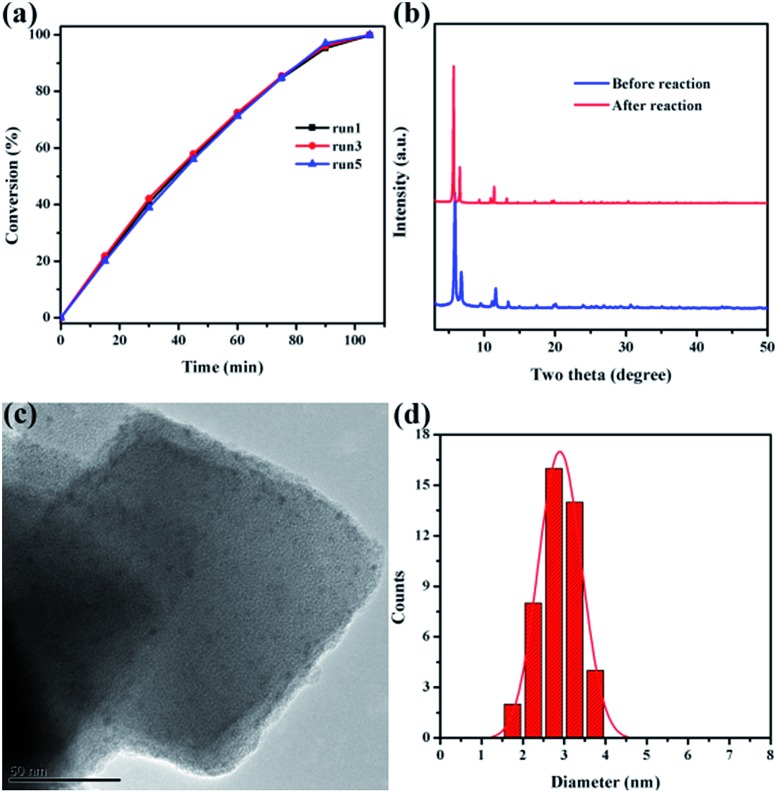
(a) Reusability of Pd@Ag-in-UiO-67 (30 : 1). (b) Powder XRD patterns of Pd@Ag-in-UiO-67 (30 : 1) before and after reaction. (c) TEM image of Pd@Ag-in-UiO-67 (30 : 1) after being used five times and (d) corresponding size distribution of Pd@Ag NPs.

Catalytic stability is of great importance for practical applications of highly efficient catalysts. After reaction completion, the catalyst was recovered from the solution and then reutilized for another reaction run under identical reaction conditions using fresh reagents. Pd@Ag-in-UiO-67 (30 : 1) showed no significant changes in terms of the catalytic activity and selectivity after successive reuses up to five cycles (Fig. 4a). The reused catalyst was also analyzed using PXRD, TEM and AAS to determine its structural and chemical stability. PXRD pattern and TEM of the reused catalyst remained virtually unchanged in terms of the structure, nanoparticle sizes and dispersion, confirming also the maintained structure of UiO-67 (Fig. 4b–d).

The doped metal content of the reused material was also confirmed to be almost identical to the fresh catalyst. Leaching experiments were eventually performed to verify the key role of Pd@Ag NPs as catalytically active species as well as to confirm the heterogeneous nature of the catalyst. With this purpose, a typical reaction was conducted using Pd@Ag-in-UiO-67 (30 : 1) as catalyst for 15 min after which the catalyst was removed from the reaction mixture by centrifugation. Upon catalyst removal, the mixture in the absence of the catalyst was left to react for several hours without any further observed increase in the conversion which strongly suggested that the reaction was heterogeneously catalyzed (Fig. S7†). These findings were consistent with AAS experiments as no Pd traces (below the detection limit, 0.5 ppm) were observed in the reaction mixture. These findings indicated that Pd@Ag-in-UiO-67 was a highly active and stable catalyst for room temperature hydrogenations under the investigation conditions, suggesting that the confinement effect offered by the framework and the strong metal–support interactions should play important roles in preventing Pd@Ag NPs from aggregation.

## Conclusions

In summary, we have successfully fabricated ultrafine core–shell bimetallic Pd@Ag NPs within the pores of MOFs *via* a seed mediated growth strategy. The deposition of Ag on Pd could separate active Pd sites and modify the electronic structure of Pd, leading to a significant increase in the selectivity in the room temperature partial-hydrogenation of phenylacetylene, selected as the model reaction. Interestingly, Ag/Pd ratios could be easily tuned in the synthesized Pd@Ag UiO-67 by controlling the addition of different amounts of AgNO_3_. This effective and unprecedented strategy reported herein could facilitate the control of the size, structure and composition of a range of metal NPs within the MOF pores for the potential design of encapsulated core–shell nanocomposites with applications in a variety of fields including heterogeneous catalysis, photocatalysis and biomass conversion, to be further reported in due course.
