# Supplementary material for: Seed-mediated growth of MOF-encapsulated Pd@Ag core–shell nanoparticles: toward advanced room temperature nanocatalysts
Source: Chem Sci. 2015 Sep 23;7(1):228–33. doi: 10.1039/c5sc02925b (PMC5515064; doi:10.1039/c5sc02925b)
Supplement: Supplementary file 1 [file SC-007-C5SC02925B-s001.pdf]

Electronic Supplementary Material (ESI) for Chemical Science.
This journal is © The Royal Society of Chemistry 2015

Supporting Information

Seed-mediated growth of MOF-encapsulated Pd@Ag core-shell nanoparticles: toward advanced room temperature nanocatalysts

Liyu Chen,[a] Binbin Huang,[a] Xuan Qiu,[a] Xi Wang,[a] Rafael Luque[b] and Yingwei Li*[a]*

[a] School of Chemistry and Chemical Engineering, South China University of Technology, Guangzhou 510640, China.

[b] Departamento de Química Orgánica, Universidad de Córdoba, Edif. Marie Curie, Ctra Nnal IV_a, Km 396, E14014, Córdoba, Spain.

* Corresponding authors. Email: liyw@scut.edu.cn (Y. L.); q62alsor@uco.es (R. L.)

Experimental

1. Catalysts preparation

All chemicals were purchased from commercial sources and used without further treatments.

1.1 Synthesis of Pd-in-UiO-67

For the synthesis of 1.0% Pd(II)-in-UiO-67: $ZrCl_4$ (70.0 mg), 2,2'-bipyridine-5,5'-dicarboxylic acid (H_2bpydc)(73.3 mg), $Pd(NO_3)_2$ (3.0 mg) and glacial acetic acid(2 mL) were dispersed in DMF(10 mL), sealed in a 25 mL tube. The slurry was subjected to ultrasounication (180 Watt) for 20 minand was then heated at 100 ºC for 30 hours. The produced powders were isolated by centrifugation, washed first with DMF (2 x 30 mL) and then with chloroform (2 x 20 mL). The sample was slowly dried in air at room temperature for 1 h, further dried under vacuum at 150 °C overnight to remove the solvents. The Pd(II)-in-UiO-67 was treated in the stream of H_2 at 200 ºC for 2 h to yield Pd-in-UiO-67.

1.2Synthesis of Pd@Ag-in-UiO-67

Pd-in-UiO-67 (50 mg) was dispersed in 15mL DMF in a 50 mL two-necked flask.H_2 gas wascontinuously bubbled though the solution for 1 h at roomtemperature under vigorousagitation. Then the H_2 gas was moved away and the systemremained to be closed. An appropriate amount of $AgNO_3$ solution was injected by aninjector under stirring.After 1 h, the reaction was stopped by ventingthe system. The products were

collected via centrifugation, washed first with DMF (2 x 30 mL) and then with chloroform (2 x 20 mL). The sample was slowly dried in air at room temperature for 1 h, further dried under vacuum at 150 °C overnight to remove the solvents.By adding different amount of $AgNO_3$ into the synthetic solution, the Ag/Pd ratio in the prepared Pd@Ag NPs can be easily tuned.

1.3Synthesis of Ag-in-UiO-67

For the synthesis of 1.0% Ag(I)-in-UiO-67: $ZrCl_4$ (70.0 mg), 2,2'-bipyridine-5,5'-dicarboxylic acid (H_2bpydc)(73.3 mg), $AgNO_3$ (2.5 mg) and glacial acetic acid (2 mL) were dispersed in DMF(10 mL), sealed in a 25 mL tube. The slurry was subjected to ultrasounication (180 Watt) for 20 minand was then heated at 100 ºC for 30 hours. The produced powders were isolated by centrifugation, washed first with DMF (2 x 30 mL) and then with chloroform (2 x 20 mL). After dried in air at room temperature for 1 h, thecollected solid was dispersed in 2 mL of DMF and was stirred for 0.5 h in an ice bath.A freshly prepared DMF solution of $NaBH_4$ (0.1 M, 1.5 mL) was added undervigorous stirring to obtain a dark brown colloidal dispersion. The suspension wasstirred at 0 °C for 5 min, followed by washing thoroughly with DMF and $CHCl_3$. Thesample was further dried under vacuum at 150 °C overnight to remove the solvents.

2. Catalyst characterization and catalytic reactions

2.1 Characterization

The BET surface area measurements were performed with N_2 adsorption isotherms at 77 K on a Micromeritics ASAP 2020M instrument. Before the analysis, the samples were evacuated at 150 °C for 12 h. Powder X-ray diffraction patterns of the samples were recorded on a Rigaku diffractormeter (D/MAX-IIIA, 3 kW) using Cu Kαradiation (40 kV, 30 mA, λ=0.1543 nm). X-ray photoelectron spectroscopy (XPS) measurements were performed on a PHI Quantera II system with a base pressure of $5x10^{-10}$ Torr. The size and morphology of Pd-MOF composites were investigated by using a transmission electron microscope (Titan G^2 60-300) with EDX analysis (Super-X) operated at 300 kV. The palladium contents of the samples were determined quantitatively by atomic absorption spectroscopy (AAS) on a HITACHI Z-2300 instrument.

2.2Catalytic reactions

Phenylacetylene (0.1 mmol) and M-in-UiO-67 catalyst (1 mol% based on Pd) were added to 2 mL of THF. The reaction mixture was stirred at room temperature under 1 atm hydrogen atmosphere. Upon reaction completion, the catalyst particles were removed from the solution by filtration and washed with THF. The liquid phase was subsequently analyzed by GC/MS (Agilent Technologies7890B-5977A GC/MSD equipped with a 0.25 mm × 30 m HP-5MS capillary column).

For the recyclability tests, the reactions were performed under the same reaction conditions as described above, except using the recovered catalyst. Each time, the catalyst was isolated from the reaction solution at the end of the reaction, washed with THF, and then dried at 150 °C under vacuum.

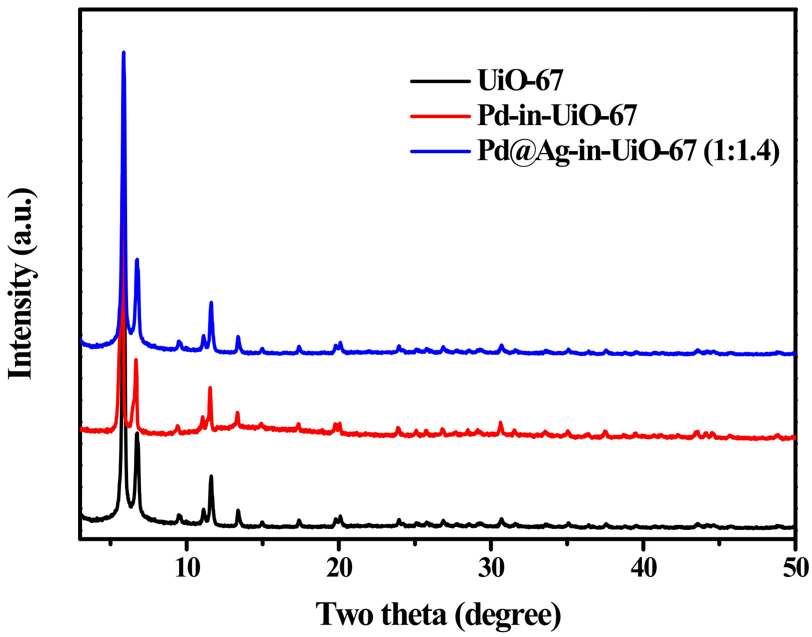

Figure S1. Powder XRD patterns of UiO-67 samples.

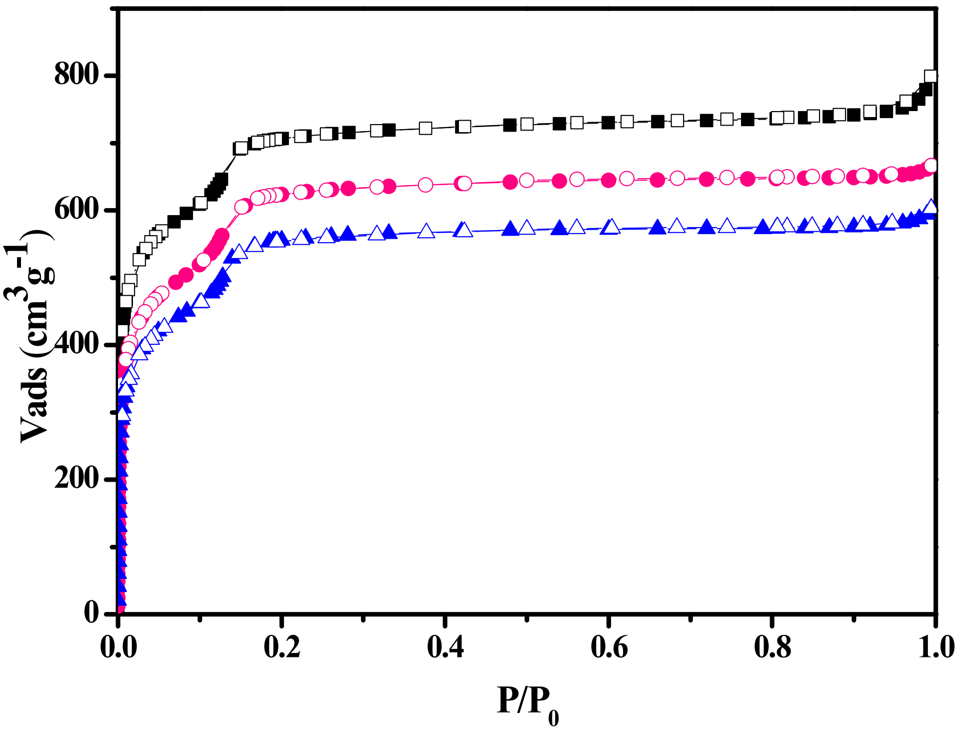

Figure S2. N_2 adsorption/desorption isotherms at 77 K for UiO-67 (■), Pd-in-UiO-67 (●), and Pd@Ag-in-UiO-67 (1:1.4) (▲). Open symbols represent the desorption branches.

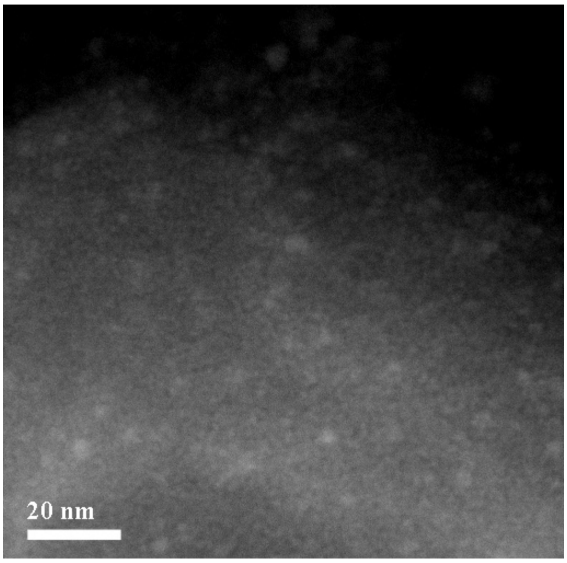

Figure S3. HAADF-STEM image of ultrathin cuts from Pd-in-UiO-67.

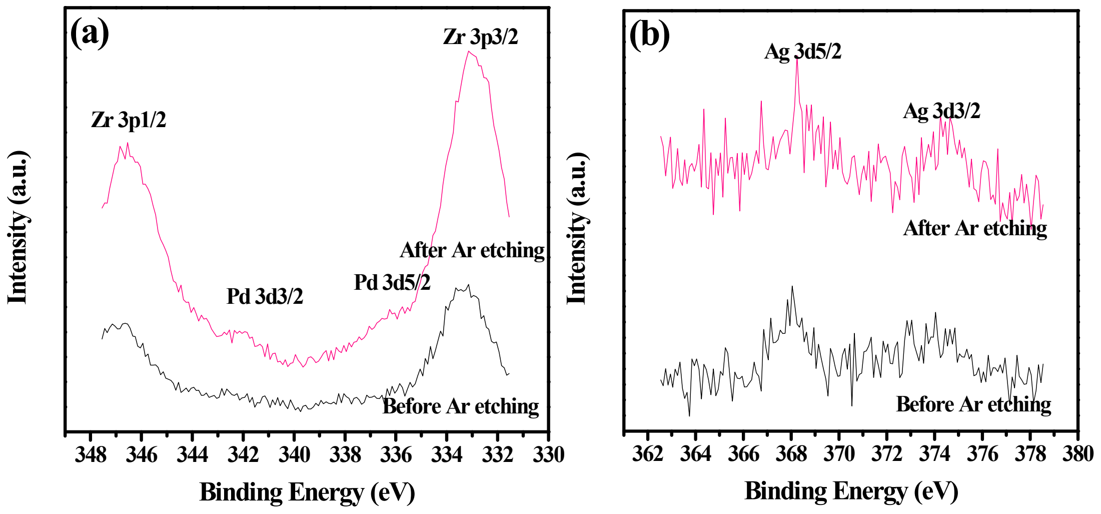

Figure S4. XPS of Pd@Ag-in-UiO-67 before and after Ar etching (10 min) at Pd 3d and Ag3d levels.

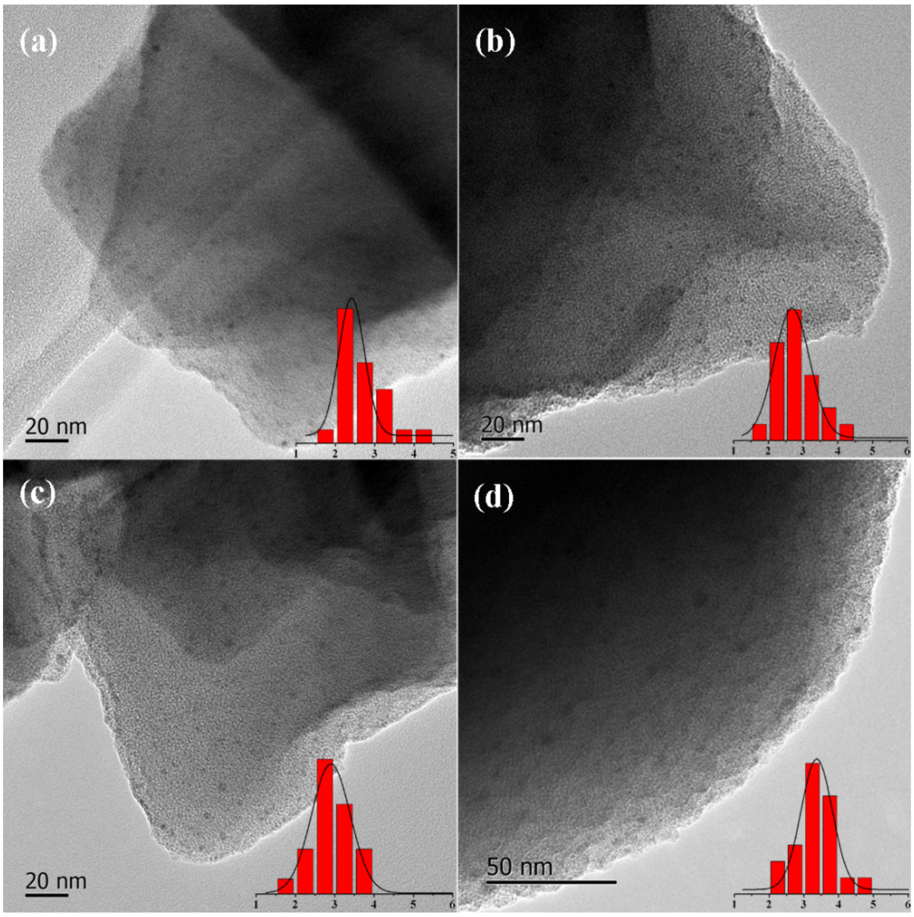

Figure S5. TEM images of the Pd@Ag-in-UiO-67 prepared by adding various amount of $AgNO_3$ into the synthetic solution: (a) 30 μg, (b) 50 μg, (c) 100 μg, and (d) 4 mg.

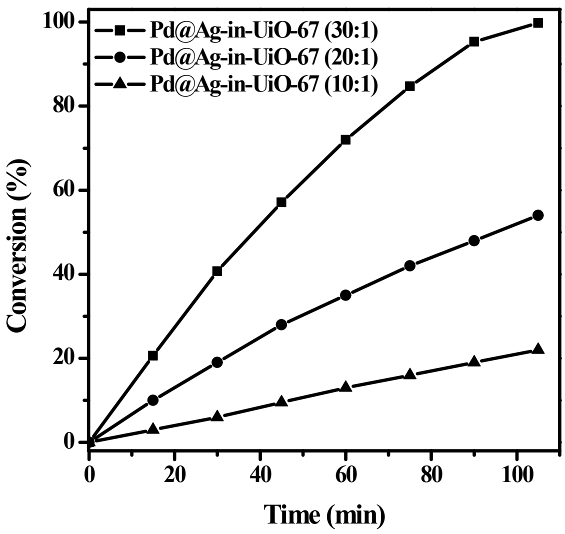

Figure S6. Activity profiles for the hydrogenation of phenylacetylene using Pd@Ag-in-UiO-67 with different Pd/Ag ratios.

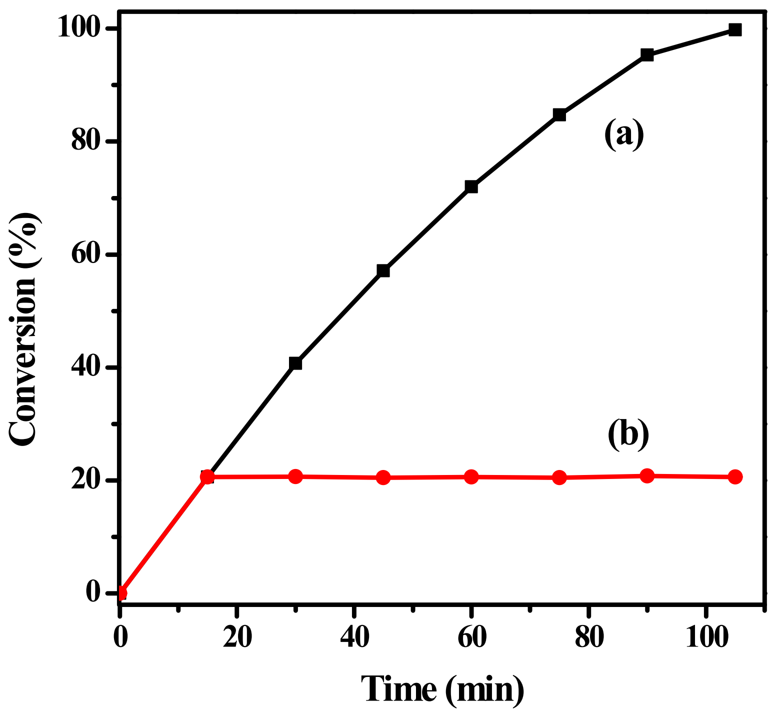

Figure S7. Activity profile for the hydrogenation of phenylacetylene. Reactionconditions: phenylacetylene (0.1 mmol), Pd@Ag-in-UiO-67 (30:1) (1% Pd), THF (2 mL), 1 atmH_2, 25 °C. (a) With catalyst, and (b) with filtrate.

Table S1. Surface areas and pore volumes of the M-in-MOF samples.

Sample	S_{BET} (m^2 g^{-1})	$S_{Langmuir}$ (m^2 g^{-1})	V_{pore} (cm^3 g^{-1})
UiO-67	2415	3082	1.10
Pd-in-UiO-67	2101	2697	0.98
Pd@Ag-in-UiO-67 (1:1.4)	1893	2438	0.87

Table S2. Pd@Ag-in-UiO-67 prepared by adding various amount of $AgNO_3$ into the synthetic solution.

Sample	$AgNO_3$ amount	Pd/Ag
Pd@Ag-in-UiO-67	30 μg	30:1
Pd@Ag-in-UiO-67	50 μg	20:1
Pd@Ag-in-UiO-67	100 μg	10:1
Pd@Ag-in-UiO-67	2 mg	1:1.4
Pd@Ag-in-UiO-67	4 mg	1:1.4
